# Effects of short-term warming and grazing on soil microbial communities in saline-alkaline grasslands of northern China

**DOI:** 10.3389/fmicb.2026.1834208

**Published:** 2026-06-12

**Authors:** Jingjing Wang, Gaoliang Pang, Peiyan Jia, Jianyu Wang, Yicong Chen, Huili Jia, Kuanhu Dong, Huajie Diao

**Affiliations:** 1 College of Grassland Science, Shanxi Agricultural University, Taigu, China; 2 Youyu Loess Plateau Grassland Ecosystem National Research Station, Shanxi Agricultural University, Youyu, China; 3 Shanxi Key Laboratory of Grassland Ecological Protection and Native Grass Germplasm Innovation, Shanxi Agricultural University, Taigu, China

**Keywords:** bacterial communities, fungal communities, grazing, saline-alkaline grasslands, warming

## Abstract

**Introduction:**

Soil microbial communities play a central role in material cycling and energy transformation within grassland ecosystems. Alterations in their community structure, diversity, and function directly influence soil ecological functions and ecosystem stability. Global warming exerts persistent environmental pressure on soil microbial communities by modifying soil water and thermal conditions. As a widespread form of human disturbance, grazing interacts with warming to potentially exert complex effects on subterranean microbial processes.

**Methods:**

This study investigates the coupled effects of short-term warming and grazing on soil bacterial and fungal communities in saline-alkaline grasslands of northern China. A factorial experiment combining canopy warming (+ 2°C) with four grazing intensities (no grazing, light grazing, moderate grazing, heavy grazing) was conducted, using high-throughput sequencing and multivariate statistical analysis.

**Results:**

The research findings indicate that the bacterial community is dominated by Pseudomonadota and Actinomycetota, while the fungal community is primarily composed of Ascomycota and Basidiomycota. The interaction between warming and heavy grazing significantly increased the relative abundance of Pseudomonadota. Warming significantly enhanced the bacterial richness index, whereas fungal α-diversity indices showed an overall declining trend under warming. Grazing tended to increase the topological complexity of bacterial networks, while decreasing that of fungal networks. The impact of warming on network topological structure was relatively limited. Pearson correlation and Redundancy Analysis (RDA) confirmed that dissolved nitrogen (DN) and the ratio of dissolved organic carbon to dissolved nitrogen (DOC/DN) were the main environmental factors driving microbial community variation in this region. Soil microbial communities were primarily associated with nutrient factors, while also being influenced by soil water content.

**Conclusion:**

Overall, the effects of grazing on soil bacterial and fungal community structure and α-diversity were relatively minor. Bacterial and fungal α-diversity indices showed differentiated responses to warming, with warming significantly increasing bacterial richness while fungal diversity declined under warming. Available nutrients (DN and DOC/DN) were the primary drivers of microbial community variation. This study extends research on the impact of short-term warming pulses against a background of long-term grazing on soil microbial communities in saline-alkaline grasslands, revealing the critical role of available nutrients in regulating microbial communities in such ecosystems. It provides a scientific reference for the adaptive management of grasslands in ecologically fragile regions of northern China under climate change.

## 1 Introduction

Soil microbial communities are fundamental components of grassland ecosystem functions, playing a crucial role in global nutrient cycling and energy transformation, and exerting a vital influence on soil ecological functions and ecosystem stability (Zhao et al., 2024). Global warming has become a major challenge facing grassland ecosystems, directly altering soil water and thermal conditions and affecting microbial metabolic processes (Zhao et al., 2024; Fu G. et al., 2024). As one of the predominant forms of anthropogenic disturbance in grasslands, grazing indirectly regulates microbial communities by modifying vegetation and the soil environment (Chen et al., 2024). Current research largely focuses on the individual effects of warming or grazing, mostly conducted in typical grassland ecosystems (Fu F. W. et al., 2024). A systematic understanding of how their interaction affects soil microbial communities in saline-alkaline grasslands, and the differential response mechanisms of bacteria and fungi, is still lacking.

Grazing activities, through livestock foraging, trampling, and excretion, alter aboveground vegetation composition, the quantity and quality of litter inputs, and soil physical structure, thereby affecting the growth microenvironment and resource availability of soil microorganisms (Zhang Y. et al., 2023; Li et al., 2024). As a primary management factor, grazing intensity typically exerts a gradient effect on soil microorganisms (Khatri-Chhetri et al., 2024). Light or moderate grazing may enhance microbial activity and diversity by increasing plant diversity and labile organic matter inputs (Qiqige et al., 2023; Xun et al., 2018). With increasing grazing intensity, microbial activity may be suppressed, leading to simplified community structure and reduced function (Yang et al., 2022; Wang et al., 2023). Due to their extensive hyphal networks and strong environmental tolerance, fungi exhibit different response patterns in response to grazing disturbances compared to rapidly growing bacteria (Zhou et al., 2023). The net effect of grazing on soil microbial communities hinges on the balance between nutrient inputs from livestock excreta and the reduction in organic matter return due to vegetation removal, a balance that shifts with grazing intensity (Eldridge et al., 2017).

The IPCC projects that under an intermediate emission scenario, global mean surface temperature will increase by 1.5–2.0°C by the mid-21st century (IPCC, 2021), and regional climate models for the agro-pastoral ecotone of northern China similarly predict a warming trend of approximately 2°C (Zhao et al., 2020). Short-term warming is a key experimental approach for simulating the early ecological effects of global warming (Hu et al., 2023). Warming primarily influences microbial communities by accelerating soil organic matter mineralization and releasing readily available nutrients, providing a resource advantage for the proliferation of copiotrophic bacteria (Meng et al., 2019; Yang et al., 2024). Simultaneously, warming intensifies surface water evaporation, potentially subjecting microorganisms to water stress and suppressing their overall activity and species diversity (Chen et al., 2023). The effects of warming on microbial communities encompass both nutrient enhancement and moisture limitation, and the net outcome is modulated by initial soil moisture, nutrient status, and the functional attributes of the microorganisms themselves (Zhao et al., 2024; Hu et al., 2023; Chen et al., 2023). Different microbial groups may respond divergently to warming: bacteria, with short generation times, can rapidly respond to nutrient pulses, whereas fungal hyphal growth and spore germination are highly dependent on soil moisture, making them potentially more vulnerable to the adverse effects of warming-induced soil water deficit.

The interaction between warming and grazing can affect soil microbial communities through two potentially opposing pathways. The first involves changes in available nutrients (a synergistic effect): warming accelerates soil organic matter mineralization, releasing dissolved organic carbon and nitrogen, while grazing provides additional nutrient inputs through livestock excreta and alters litter quality. Their superposition can synergistically enhance nutrient supply for microbial growth. The second pathway involves the superimposition of water stress (an antagonistic effect): warming intensifies surface evaporation, reducing soil moisture, while heavy grazing further impairs soil water retention capacity through compaction by trampling. The combined water stress can exert a stronger constraint on microbial communities, especially for moisture-sensitive groups like fungi (Usman et al., 2024; Qi et al., 2024). The net outcome of this interaction depends on the relative strength of enhanced nutrient availability versus amplified stress, and may vary with grazing intensity, microbial group, and local environmental conditions. When increased nutrient supply outweighs environmental stress, the two may synergistically promote microbial growth; when water limitation dominates, the positive effects of nutrient release may be partially or completely offset, exhibiting an antagonistic character (Yuan et al., 2021; Zhao et al., 2025). At the network level, changes in habitat heterogeneity caused by grazing and resource fluctuations induced by warming may reshape potential associations among microbial taxa, thereby affecting the topological complexity of co-occurrence networks. Furthermore, copiotrophic bacteria tend to utilize resources with lower C:N ratios (Qiang et al., 2024), and shifts in substrate stoichiometry under the combined influence of warming and grazing may further mediate changes in community composition.

The agro-pastoral transition zone in northern Shanxi represents a typical ecologically fragile region in northern China (Qiao et al., 2024), where saline-alkaline constitutes one of the primary forms of grassland degradation (Diao et al., 2024; Zhang G. L. et al., 2023; Wei et al., 2024a). Such saline-alkaline soils are characterized by high pH (9–10), high electrical conductivity, and stoichiometric imbalances, prominently featuring low nitrogen availability relative to carbon. Prolonged exposure to these extreme conditions imposes strong selective pressure on soil microbial communities, retaining taxa with high tolerance to osmotic stress and alkalinity while also rendering community structure particularly sensitive to changes in resources such as water and available nutrients. This study focuses on the saline-alkaline grasslands of northern Shanxi. Through a factorial experiment combining grassland canopy warming and different grazing intensities, along with high-throughput sequencing and multivariate statistical analysis, we aim to investigate the coupled effects of short-term warming and grazing on soil bacterial and fungal communities. The study addresses the following questions: (1) How do grazing, warming, and their interaction alter the composition and diversity of soil bacterial and fungal communities in saline-alkaline grasslands? (2) What are the key environmental factors driving changes in soil microbial communities? (3) How does the legacy effect of long-term grazing modulate microbial community responses to short-term warming? In this study, grazing treatments have been applied for 8 years, while warming was implemented for 1 year, creating a temporal superimposition of a long-term background and a short-term pulse. This design facilitates an exploration of whether long-term grazing, through legacy effects, has modulated the microbial community’s response to short-term warming, while also providing a theoretical reference for the adaptive management of grasslands in ecologically fragile regions of northern China under climate change.

## 2 Materials and methods

### 2.1 Experimental site description

The experimental site was established based on the National Field Scientific Observation and Research Station of the Loess Plateau Grassland Ecosystem in Youyu, Shanxi Province (112° 19’E, 39° 59’N), with an altitude of 1,348 m. The region has a temperate semi-arid continental climate. According to long-term meteorological observation data (1989–2019), the average annual temperature is 4.6°C, the average annual precipitation is 425 mm, and the precipitation is mostly concentrated from June to August, accounting for 59% of the total annual amount. The vegetation growth season is from May to September, with peak growth in mid-August. The study site is a moderately degraded saline-alkaline grassland, with vegetation coverage within the experimental area ranging from 40 to 60% and a mean peak aboveground biomass of approximately 180 g⋅m^–2^ (Hao et al., 2024). Dominant plant include *Leymus secalinus* (Georgi) Tzvelev, *Puccinellia distans* (Jacq.) Parl., and *Artemisia anethifolia Weber* ex Stechmann. The soil is chestnut calcareous soil (Chinese classification), with a pH of 9–10 and distinct saline-alkaline characteristics.

### 2.2 Experimental design

This study employed a randomized block design (Supplementary Figure 1), with two experimental factors: grazing intensity and warming treatment. Grazing intensity was set at four levels based on local grassland productivity and carrying capacity: no grazing (0 sheep⋅hm^–2^, NG), light grazing (2.35 sheep⋅hm^–2^, LG), moderate grazing (4.80 sheep⋅hm^–2^, MG), and heavy grazing (7.35 sheep⋅hm^–2^, HG). The light, moderate, and heavy grazing stocking rates corresponded to approximately 50%, 100%, and 150% of the recommended moderate stocking rate for this region, respectively (Diao et al., 2024). Warming treatment was set at two levels: ambient control (CK) and canopy warming (+ 2°C, W). Within each block, four main plots were randomly assigned to the four grazing intensity levels. Each main plot covered an area of 2,000 m^2^ (100 m × 20 m) and was enclosed by a wire fence (1.5 m height) for grazing management. Within each grazing main plot, warming and control subplots were randomly nested. Thus, 8 treatment combinations (4 grazing intensities × 2 warming treatments) were replicated 4 times each, for a total of 32 experimental subplots. The grazing livestock were Dorset ewes. Grazing disturbances commenced in 2017 and were carried out annually from June 1 to September 30, resulting in a cumulative grazing duration of 8 years.

An open-top infrared warming system (SY-FATI, National Energy Group Electric Power Research Institute Co., Ltd., China), designed according to the Temperature Free-Air Controlled Enhancement (T-FACE) principle (Supplementary Figure 2). The heating device had a power of 800 W (220 V) and was suspended in a triangular array at a height of 1.5 m above the ground, covering a heating area of approximately 9 m^2^. Both warmed and control plots were equipped with temperature sensors, and an automatic feedback control system continuously regulated heater output to maintain a + 2°C difference in vegetation canopy temperature between warmed and corresponding control plots throughout the growing season (Kimball et al., 2008). The heating system was installed in May 2023 and operated annually from May to September. Sampling occurred in August 2024, representing a cumulative heating period of 1 year.

### 2.3 Soil sampling and physicochemical property analysis

Soil samples were collected in August 2024. In each subplot, six soil cores (0–10 cm depth) were randomly collected from a core sampling area (approximately 4–5 m^2^) using a 5 cm diameter soil auger and composited into one sample. The samples were sieved through a 2 mm mesh to remove impurities such as roots and stones. The total number of composite soil samples for final analysis was 32 (4 blocks × 4 grazing intensities × 2 warming treatments). Each sieved sample was divided into three portions: one was air-dried at room temperature and stored at room temperature for measuring soil pH and electrical conductivity (EC); one was refrigerated at 4°C for the determination of dissolved organic carbon (DOC) and dissolved nitrogen (DN); and one portion is stored at -80°C for subsequent high-throughput DNA sequencing.

Soil water content (SWC) was measured monthlyat the beginning and end of each month from May to August using a portable soil moisture meter (TDR-350, Spectrum Technologies, United States) in each subplot. The SWC value used in statistical analyses was the mean of these measurements over the growing season. A 10 g air-dried soil sample was mixed with 25 mL distilled water (soil:water ratio 1:2.5), shaken thoroughly on a reciprocating shaker, and allowed to stand for 30 min to obtain the supernatant. The pH of the supernatant was measured using a pH meter (FE20, Mettler Toledo, Switzerland), and subsequently, EC was measured using a conductivity meter (DDS-307, INESA, China). For the determination of DOC and DN concentrations: water ratio of 1:2.5. Ten grams of refrigerated soil was mixed with 25 mL of extractant, shaken for 1 h, and allowed to stand for 30 min. The mixture was then filtered through quantitative filter paper into a 15 mL centrifuge tube and stored at –20°C. Samples were ultimately analyzed using a total organic carbon analyzer (Elementar Vario, Germany).

### 2.4 Soil microbial DNA extraction and high-throughput sequencing

Extract total DNA from soil samples using the E.Z.N.A.^®^ SoilDNA Kit. Assess DNA integrity via 1% agarose gel electrophoresis and determine concentration and purity (A260/A280) using the NanoDrop2000.

Bacterial 16S rRNA gene V3–V4 region amplification primers: 338F (5’-ACTCCTACGGGAGGCAGCAG-3’) and 806R (5’-GGACTACHVGGGTWTCTAAT-3’) (Xue et al., 2023); The primers for amplifying the fungal ITS1 region were ITS1F (5’-CTTGGTCATTTAGAGGAAGTAA-3’) and ITS2R (5’-GCTGCGTTCTTCATCGATGC-3’) (Bi et al., 2021). To distinguish different samples, an 8-base barcode sequence was introduced at the 5’ end of the forward primer. The PCR reaction system (20 μL) included: 10 ng template DNA, 0.4 μL TransStart Fast Pfu DNA Polymerase, 0.8 μL forward and reverse primers (5 μM), 2 μL dNTPs (2.5 mM), and 4 μL 5 × Fast Pfu Buffer. The amplification program was set as follows: 95°C pre-denaturation for 3 min; 27 cycles of 95°C for 30 s, 55°C for 30 s, and 72°C for 30 s; followed by a final extension at 72°C for 10 min. Amplification products were detected via 2% agarose gel electrophoresis, purified using a DNA gel recovery kit, and precisely quantified with Qubit 4.0. Purified products were library-prepared using the NEXTFLEX Rapid DNA-Seq Kit, encompassing adapter ligation, fragment selection, library amplification, and purification. Constructed libraries underwent 2 × 150 bp paired-end sequencing on the Illumina NextSeq 2000 platform, performed by Shanghai Meiji Biotechnology Co., Ltd.

Raw sequencing data underwent bioinformatics preprocessing: fastp (v0.19.6) performed quality filtering to remove low-quality sequences and adapters; FLASH (v1.2.11) assembled paired-end reads. Subsequently, DADA2 within the QIIME2 workflow (version 2022.11) performed denoising to generate amplificon sequence variants (ASVs). To eliminate the impact of sequencing depth variation on diversity analysis, the number of sequences per sample was normalized to 20,000 (Li et al., 2022). Species annotation of ASVs was performed using the q2-feature-classifier plugin based on the Silva138 database. Functional composition of the bacterial community was further predicted using PICRUSt2 software.

### 2.5 Statistical analysis

Bioinformatics data were exported and analyzed on the Majorbio Cloud Platform (https://cloud.majorbio.com). After removing low-quality reads with Trimmomatic, raw sequences were clustered into Amplicon Sequence Variants (ASVs) at 97% similarity using USEARCH.

Multivariate analysis of variance (MANOVA) was used to assess the overall effects of grazing intensity, warming treatmen, and their interaction (G × W) on these multivariate indicators. Subsequently, two-way ANOVA was conducted for each univariate variable, followed by *post hoc* multiple comparisons using the Least Significant Difference (LSD) method to determine significant differences among different grazing intensity levels. Permutational multivariate analysis of variance (PERMANOVA) based on Bray-Curtis distance was used to assess the effects of grazing intensity, warming treatment, and their interaction on bacterial and fungal community structure (at the genus level). Non-metric multidimensional scaling (NMDS) was used for two-dimensional ordination visualization of community structure (at the genus level). The above analyses were performed in the R language environment (R version 4.0.3), using the vegdist function in the vegan package (version 2.5-7) to calculate the Bray-Curtis distance matrix, the adonis2 function for PERMANOVA (permutations = 999), and the metaMDS function for NMDS ordination. Redundancy Analysis (RDA) was performed on the Majorbio Cloud Platform based on genus-level community composition data to assess the explanatory power of the aforementioned environmental factors on bacterial and fungal community structural variation. Prior to RDA, variance inflation factors (VIF) were examined to test for multicollinearity among environmental variables.

Soil microbial co-occurrence networks were constructed based on Spearman rank correlations to reveal changes in microbial co-occurrence patterns. Network construction was based on genus-level ASV abundance data. Before network construction, the ASV table was filtered by total abundance, removing ASVs with a total relative abundance sum across all samples below 0.01%. The same filtering criteria were applied to both bacterial and fungal datasets. The corr.test function in the psych package (version 2.1.9) in R was used to calculate Spearman correlation coefficients between genus-level ASVs, and the Benjamini-Hochberg method was applied to correct the raw *P*-values for false discovery rate (FDR). Only correlations meeting both of the following criteria were retained for network construction: (1) absolute Spearman correlation coefficient |r| > 0.8; (2) *P* < 0.01. Co-occurrence networks were visualized in Gephi software (version 0.9.2), and topological parameters such as number of nodes, number of edges, average degree, average clustering coefficient, and average path length were calculated. Node colors in the network diagrams represent taxonomic information at the phylum level. At the ASV level, α-diversity index-related data for bacterial and fungal communities, including Chao1 richness index, Shannon diversity index, Simpson index, and Pielou evenness index, were extracted from the platform. Pearson correlation analysis was used to evaluate the relationship between soil environmental factors and ASV-level microbial α-diversity indices, performed using the corr.test function in R, and results were presented as a heatmap. Other graphics were plotted in Origin 2023. The significance level for all statistical analyses was set at *P* < 0.05.

## 3 Results

### 3.1 Effects of warming and grazing on soil physicochemical properties

**FIGURE 1:**
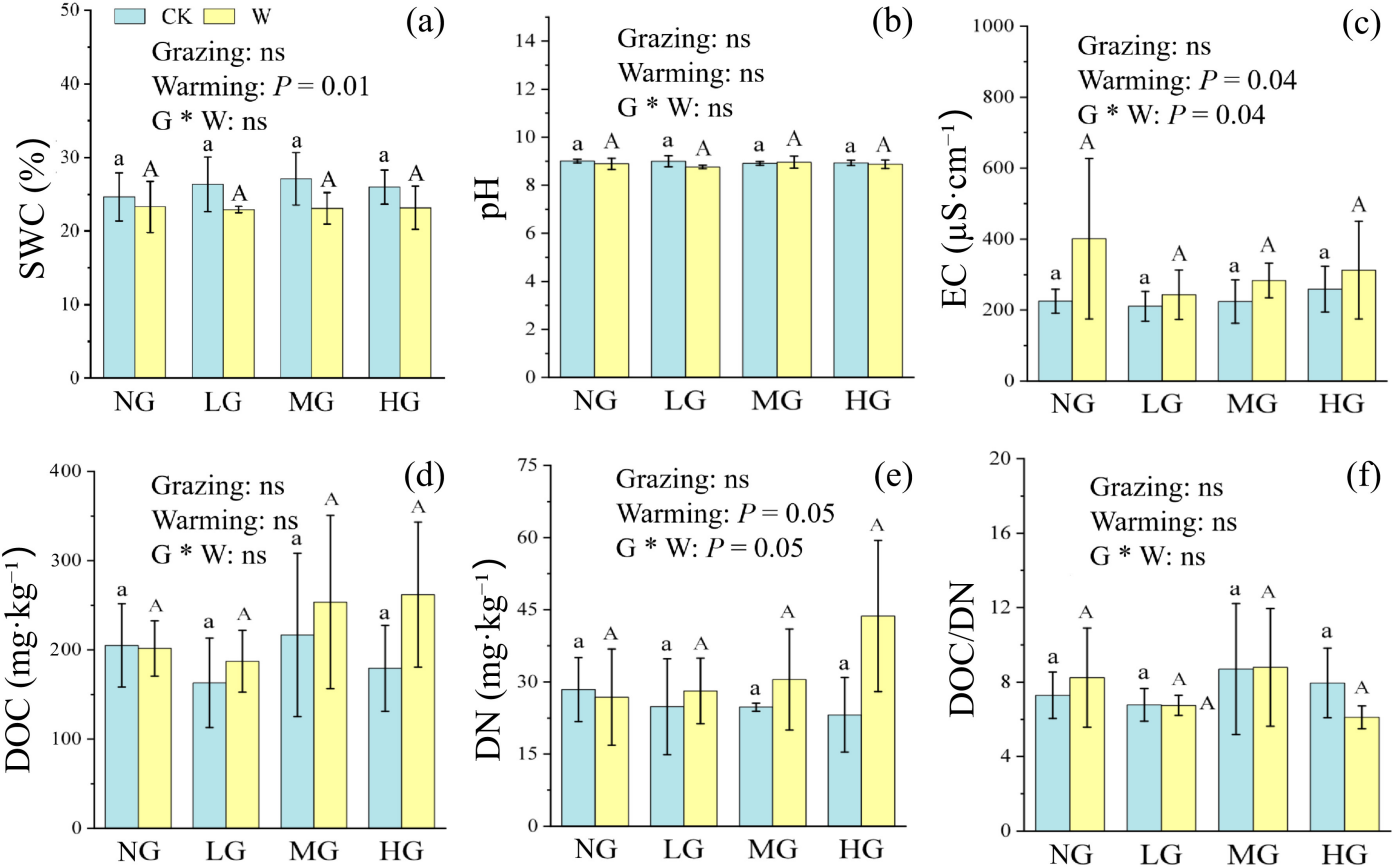
Effects of grazing and warming on soil physicochemical properties **(a–f)**. Bars represent means ± SE, *n* = 4 independent replicate plots. Lowercase letters **(a, b, c)** indicate statistically significant differences (*P* < 0.05) among different grazing intensities in the control treatment, while uppercase letters (A, B, C) denote differences among different grazing intensities under warming treatment. SWC, soil water content; EC, electrical conductivity; DOC, dissolved organic carbon; DN, dissolved nitrogen; NG, no grazing; LG, light grazing; MG, moderate grazing; HG, heavy grazing; CK, control; W, warming; G, grazing; *, interaction.

Analysis of soil physicochemical properties revealed that warming, grazing, and their interaction had varying degrees of influence on different soil parameters(Figure 1). Concurrently, warming significantly decreased SWC (*P* = 0.04, Figure 1a). At the same time, warming significantly increased EC (*P* = 0.04), and this effect was more pronounced under the no grazing condition (Figure 1c), With increasing grazing intensity, warming led to a progressive increase in soil DOC and DN content. DN showed a marginally significant response to the warming treatment and the interaction between warming and grazing (*P* = 0.05) (Figure 1e). suggesting that the interaction between warming and grazing may have promoted the release of available nutrients. Grazing intensity alone had no significant effect on soil physicochemical properties (Figures 1a–f), indicating that grazing disturbance alone may be insufficient to alter the fundamental soil physicochemical properties of this saline-alkaline ecosystem.

### 3.2 Effects of warming and grazing on microbial community composition and structure

**FIGURE 2:**
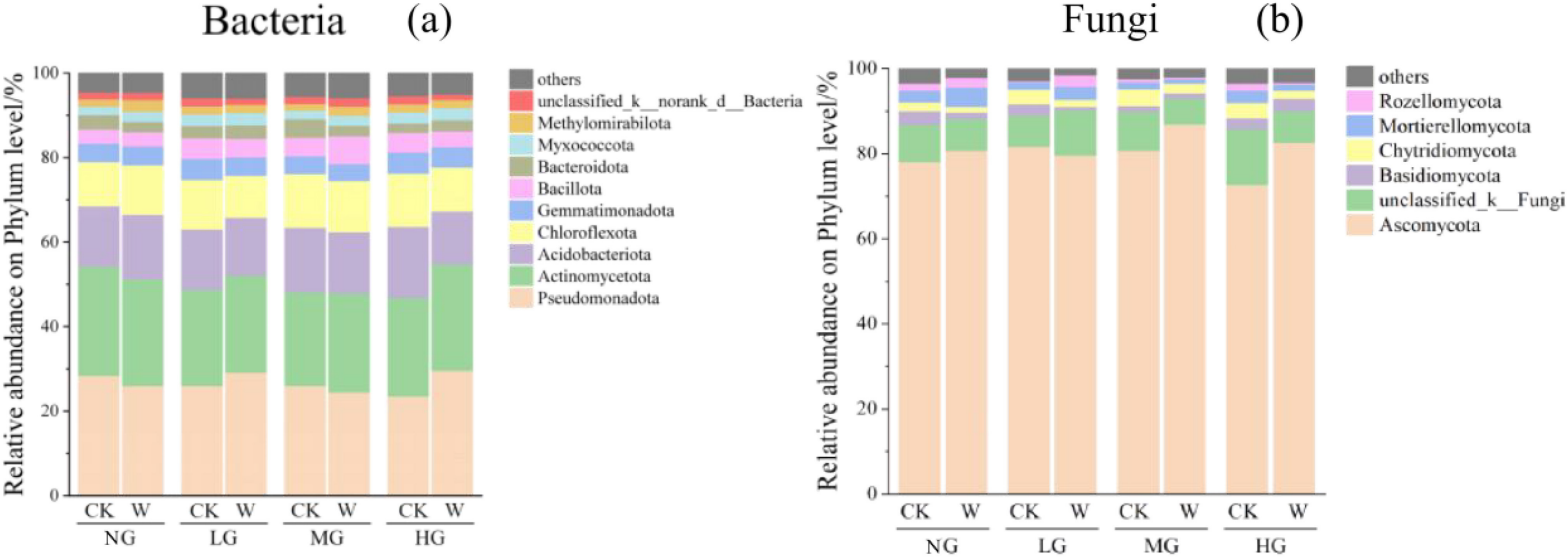
Effects of grazing and warming on the phylum-level composition of soil bacterial **(a)** and fungal **(b)** communities (*n* = 4 independent replicate plots). NG, no grazing; LG, light grazing; MG, moderate grazing; HG, heavy grazing; CK, control; W, warming.

At the phylum level, bacterial communities were primarily composed of Pseudomonadota, Actinomycetota, Acidobacteriota, and Chloroflexota (Figure 2a), while fungal communities were dominated by Ascomycota and Basidiomycota (Figure 2b). Under high grazing intensity, warming significantly increased the relative abundance of Pseudomonadota (*P* < 0.05, Supplementary Table 1). Compared with the no grazing treatment, light grazing significantly increased the relative abundance of several low-abundance bacterial phyla (classified as “others”) (Supplementary Table 2). Neither warming nor grazing treatments significantly affected the relative abundance of fungal phyla (Figure 2b and Supplementary Table 2).

**FIGURE 3:**
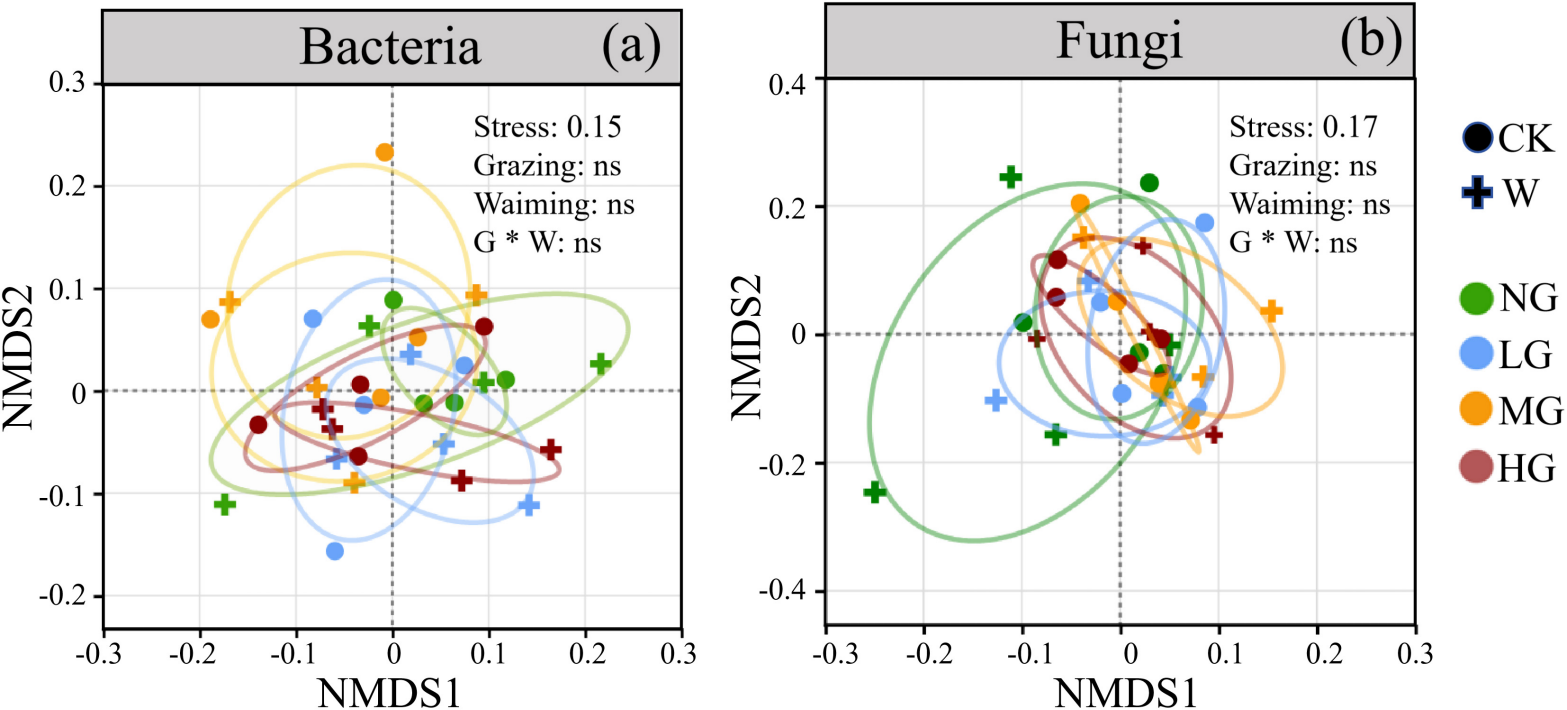
Effects of grazing and warming on the community structure of bacteria **(a)** and fungi **(b)** (*n* = 4 independent replicate plots). NG, no grazing; LG, light grazing; MG, moderate grazing; HG, heavy grazing; CK, control; W, warming; G, grazing; *, interaction.

Results from Non-metric Multidimensional Scaling (NMDS) based on Bray-Curtis distance revealed that, regardless of grazing or warming treatment, bacterial and fungal communities did not form distinct clusters in the two-dimensional ordination space, with a high degree of overlap in community structure among treatment groups (Figure 3). PERMANOVA further confirmed that warming, grazing, and their interaction had no significant effect on the β-diversity of either bacterial or fungal communities (Supplementary Table 3).

### 3.3 Effects of warming and grazing on microbial community α-diversity

**FIGURE 4:**
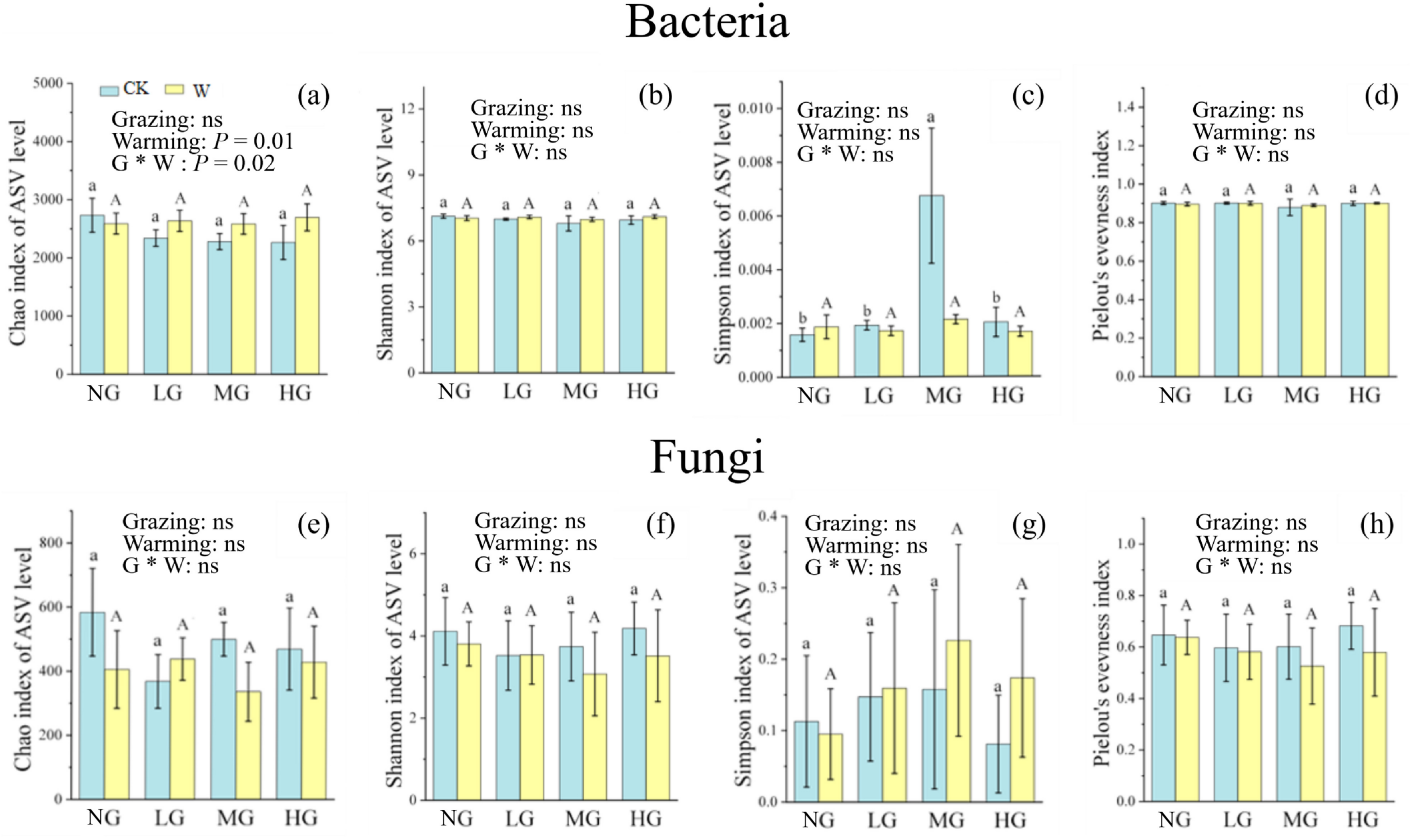
Effects of grazing and warming on the α-diversity of bacterial **(a–d)** and fungal **(e–h)** communities (*n* = 4 independent replicate plots). Bars represent means ± SE. Different lowercase letters (a, b, c) indicate statistical differences among grazing intensities within the ambient control treatment at *P* < 0.05, and different uppercase letters (A, B, C) indicate differences among grazing intensities within the warming treatment. NG, no grazing; LG, light grazing; MG, moderate grazing; HG, heavy grazing; CK, control; W, warming; G, grazing; *, interaction.

Analysis of α-diversity indices revealed different responses of bacterial and fungal communities to warming and grazing treatments (Figure 4). Warming significantly increased the Chao richness index of bacteria (*P* = 0.01), and this effect was particularly pronounced in grazed plots (LG, MG, and HG) (Figure 4a). Furthermore, warming and grazing exhibited a significant synergistic interaction on the bacterial Chao index (*P* = 0.02, Figure 4a). In contrast to bacteria, the diversity indices of fungal communities showed an overall downward trend under warming treatments, suggesting that climate warming may have an adverse effect on fungal community diversity. Grazing intensity itself did not significantly affect the α-diversity indices of bacteria or fungi (Figures 4a–h), indicating that grazing disturbance has a minor impact on the α-diversity of microbial communities in this ecosystem.

### 3.4 Effects of warming and grazing on microbial co-occurrence network topology

**FIGURE 5:**
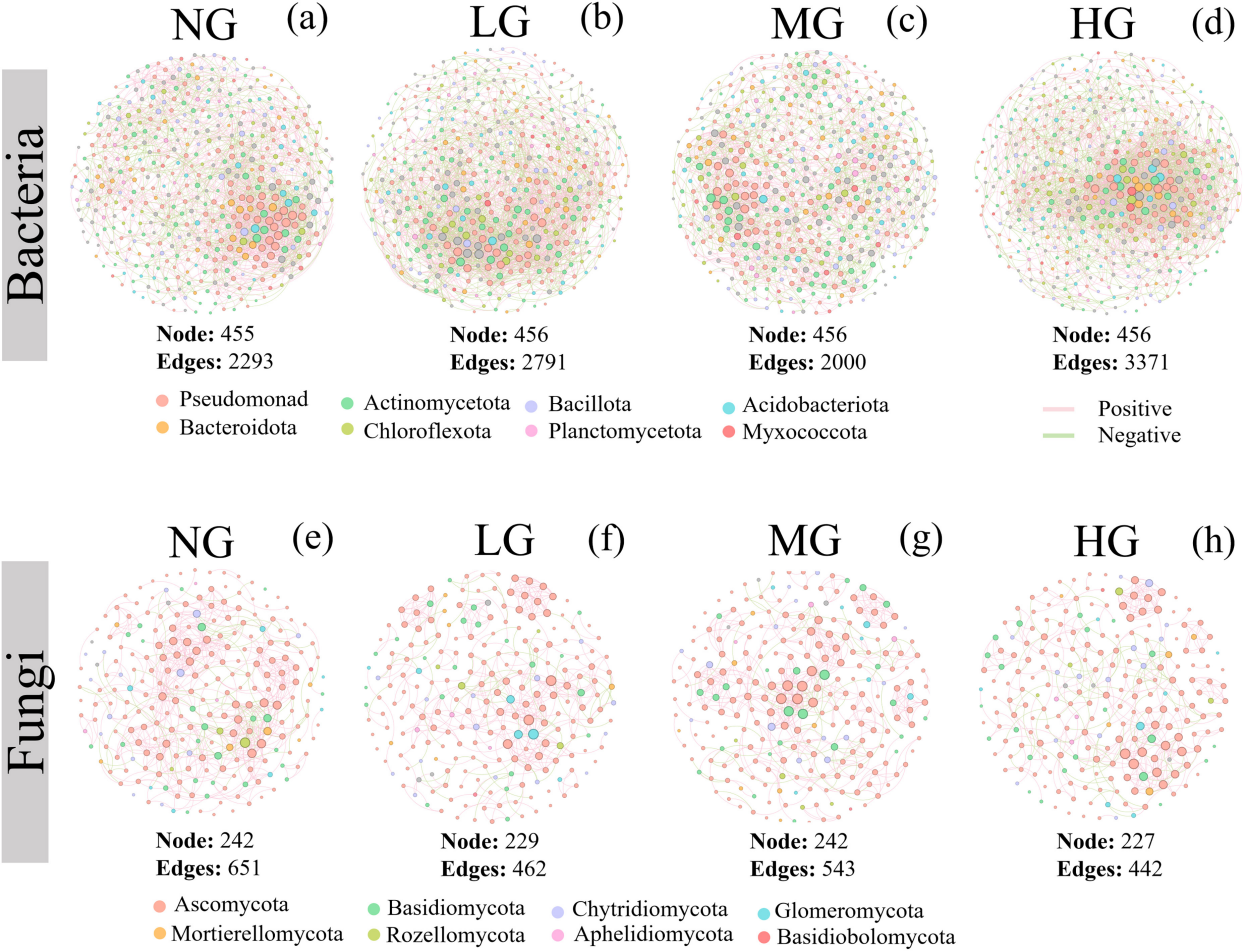
Effects of different grazing intensities on the topological structure of soil bacterial **(a–d)** and fungal **(e–h)** co-occurrence networks (*n* = 4 independent replicate plots). Nodes of different colors in the co-occurrence networks represent different microbial taxa at the phylum level. Networks were constructed based on Spearman correlation coefficients (| *r*| > 0.8, *P* < 0.01) with node size proportional to degree; red edges represent positive correlations between nodes, and green edges represent negative correlations. NG, no grazing; LG, light grazing; MG, moderate grazing; HG, heavy grazing.

Grazing intensity had divergent effects on the topological structure of bacterial and fungal co-occurrence networks (Figure 5 and Supplementary Table 4). The bacterial network under heavy grazing exhibited the highest number of nodes, edges, and average degree. The number of nodes and edges was also higher under light grazing compared to no grazing, but decreased under moderate grazing. All grazing treatments resulted in a reduction in the number of edges in fungal networks, while the average clustering coefficient increased under grazing treatments. Additionally, the proportion of positive edges in fungal networks was generally lower than in bacterial networks.

**FIGURE 6:**
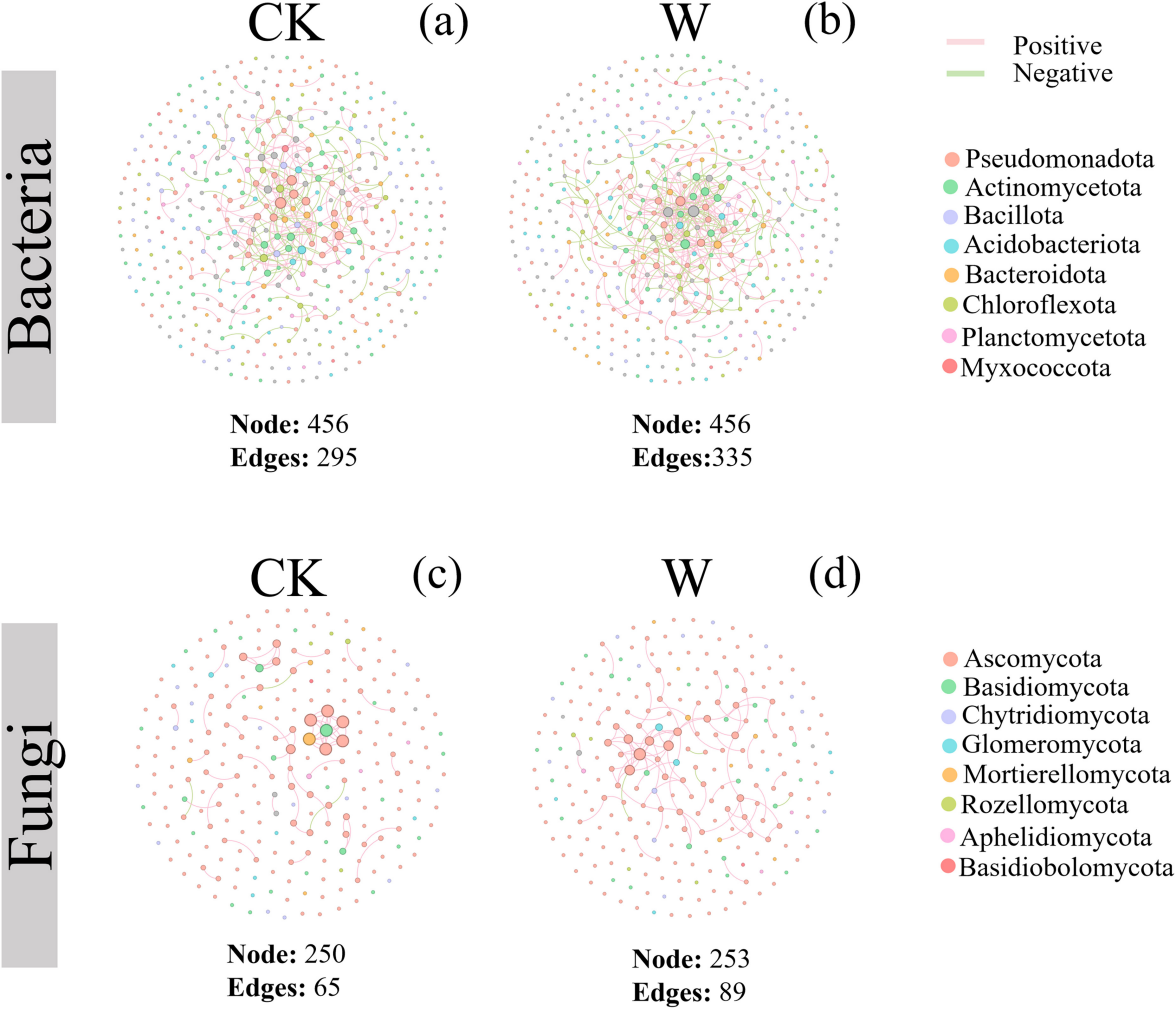
Effects of warming on the topological structure of soil bacterial **(a,b)** and fungal **(c,d)** co-occurrence networks (*n* = 4 independent replicate plots per treatment combination). Nodes of different colors in the co-occurrence networks represent different microbial taxa at the phylum level. Networks were constructed based on Spearman correlation coefficients (| *r*| > 0.8, *P* < 0.01), with node size proportional to degree; red edges represent positive correlations between nodes, and green edges represent negative correlations. CK, control; W, warming.

The effect of warming on the topological structure of bacterial and fungal networks was relatively limited (Figure 6 and Supplementary Table 4). Under warming, the bacterial network showed a slight increase in the number of edges and average degree, while the number of nodes remained largely unchanged, and the ratio of positive to negative edges did not change markedly. The fungal network under warming exhibited an increase in both the number of nodes and edges, while the average clustering coefficient decreased and the average path length increased.

Overall, the topological structure of both bacterial and fungal co-occurrence networks showed a greater magnitude of response to grazing disturbance than to warming treatment, and the direction of grazing influence was opposite for bacteria and fungi—bacterial network topological complexity tended to increase, while that of fungi tended to decrease.

### 3.5 Relationship between environmental factors and microbial communities

**FIGURE 7:**
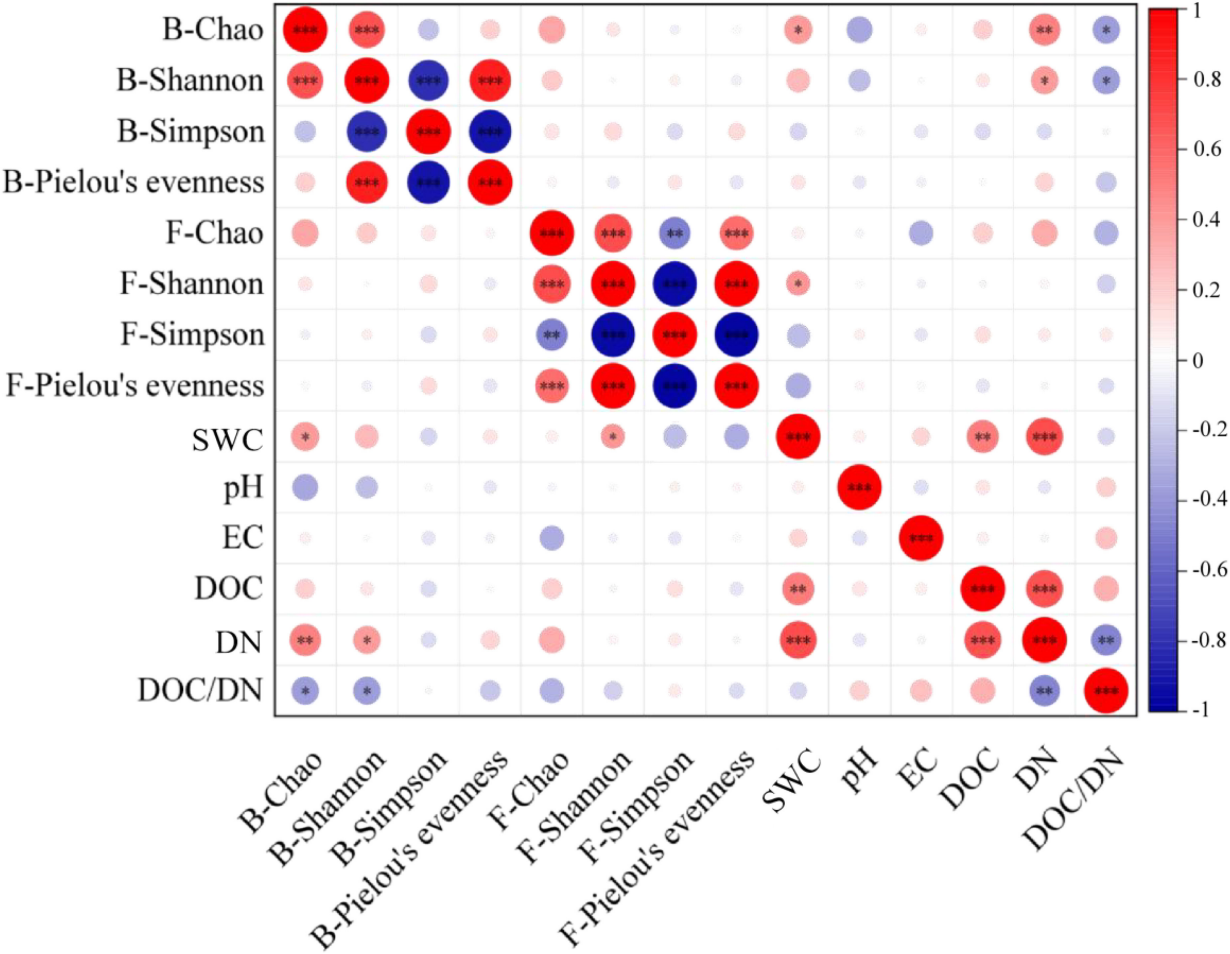
Relationships between the α-diversity of soil bacterial and fungal communities and soil physicochemical factors. The prefix “B” in α-diversity indices denotes bacteria, and “F” denotes fungi. SWC, soil water content; EC, electrical conductivity; DOC, dissolved organic carbon; DN, dissolved nitrogen, with asterisks indicating significant levels, *, **, *** representing *P* < 0.05, *P* < 0.01, *P* < 0.001.

**FIGURE 8:**
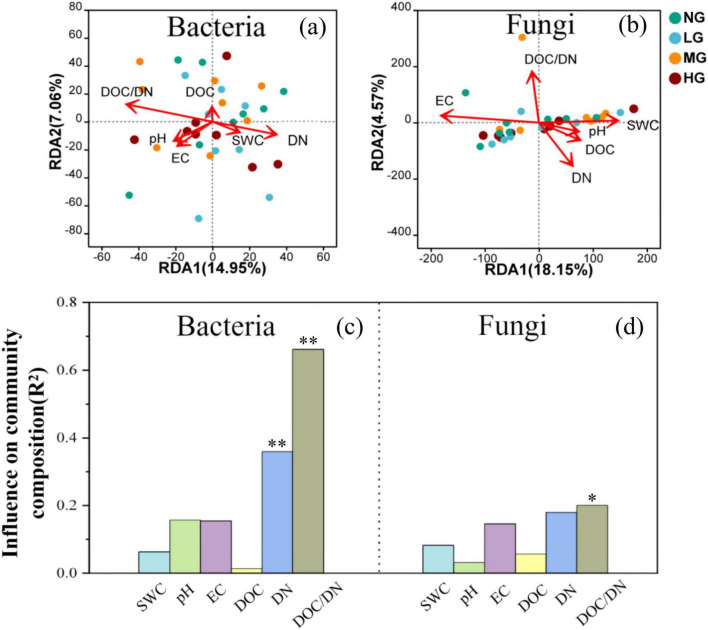
Effects of environmental variables on the variation of bacterial **(a, c)** and fungal **(b, d)** communities. NG, no grazing; LG, light grazing; MG, moderate grazing; HG, heavy grazing; SWC, soil water content; EC, electrical conductivity; DOC, dissolved organic carbon; DN, dissolved nitrogen, with asterisks indicating significant levels, *, ** representing *P* < 0.05, *P* < 0.01.

**FIGURE 9:**
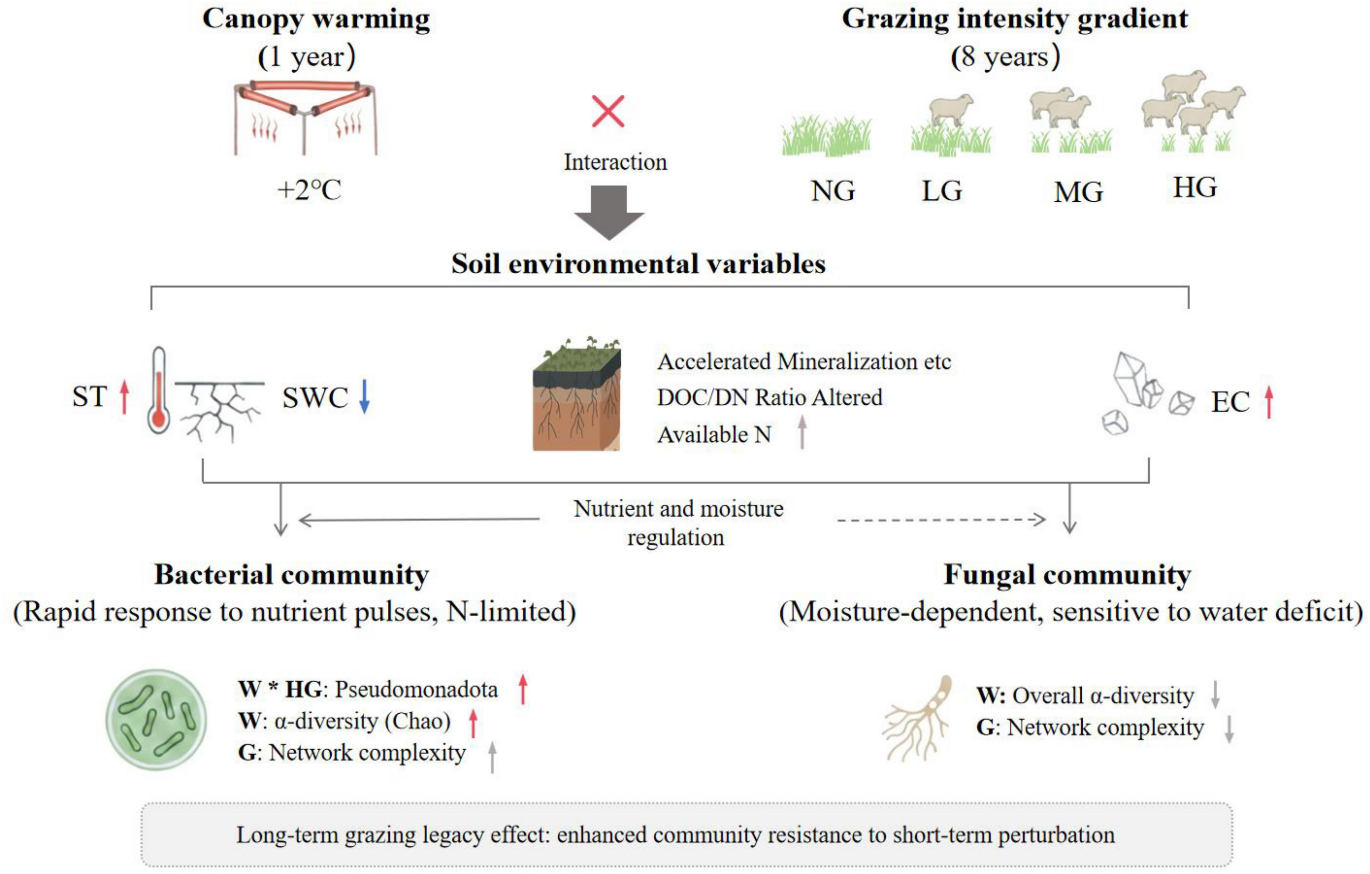
Conceptual framework illustrating the effects of grazing and warming on soil microbial communities. Upward arrows indicate increases in the indicator, and downward arrows indicate decreases. Gray arrows indicate trends of change that did not reach statistical significance, while arrows in other colors indicate statistically significant changes (*P* < 0.05). NG, no grazing; LG, light grazing; MG, moderate grazing; HG, heavy grazing; ST, soil temperature; SWC, soil water content; EC, electrical conductivity; DOC, dissolved organic carbon; DN, dissolved nitrogen; W, warming; G, grazing; *, interaction.

Pearson correlation analysis indicated that the bacterial Chao richness index and Shannon diversity index were significantly positively correlated with DN and significantly negatively correlated with DOC/DN ratio. Bacterial diversity increased with available nitrogen availability, but decreased under stoichiometric imbalance when carbon became excessive relative to nitrogen. Both the bacterial Chao index and the fungal Shannon index were positively correlated with SWC (Figure 7), indicating that SWC plays a significant role in modulating changes in the α-diversity of bacterial and fungal communities. Redundancy analysis (RDA) results further confirmed that DOC/DN and DN content are the primary environmental factors explaining changes in bacterial and fungal community structure (Figures 8c,d). The high contribution of nutrient factors indicates that, compared to basic soil variables such as pH, EC, or SWC, available nutrients exert a stronger regulatory influence on changes in microbial communities in saline-alkaline grasslands. Based on these findings, we propose a conceptual framework (Figure 9) that highlights the regulation of microbial community changes by soil water content and available nutrients.

## 4 Discussion

### 4.1 Limited response of soil bacterial and fungal community structure in saline-alkaline habitats

The NMDS and PERMANOVA results of this study indicate that neither short-term warming nor varying intensities of grazing disturbance caused significant shifts in the structure of soil bacterial and fungal communities, with samples from all treatment groups overlapping highly in the ordination space (Fu F. W. et al., 2024; Rong et al., 2022; Bi et al., 2021). The soil at the study site has typical saline-alkaline characteristics, with pH consistently maintained within a high range of 9–10, accompanied by high electrical conductivity stress. During long-term ecological succession, such significant environmental pressure has selected for and retained microbial taxa with high tolerance to salinity, osmotic stress, and desiccation (Li et al., 2021; Zhang et al., 2024), while environmentally sensitive species have been gradually eliminated (Rath et al., 2019; Oren, 2008; Wei et al., 2024a). Compared to the long-term saline-alkaline stress, the environmental pressure induced by the warming and grazing disturbances in this experiment was relatively minor and may not have reached the critical threshold required to trigger significant shifts in community structure (Zhang Y. et al., 2023; Jansson and Hofmockel, 2020; Fierer, 2017).

It is noteworthy that the experimental design of this study represents a temporal superimposition of a long-term grazing background and a short-term warming pulse. Eight consecutive years of grazing may have already imposed strong environmental filtering on the soil microbial community, selecting for taxa with broader niches and higher disturbance tolerance. This legacy effect may partially explain why no significant change in community structure was observed following the subsequent 1-year warming treatment, as the community may exhibit a degree of resistance to additional short-term perturbations (Hawkes and Keitt, 2015).

### 4.2 Differential responses of bacteria and fungi to warming and grazing

This study found that bacterial and fungal communities exhibited contrasting response patterns to warming treatment. Warming significantly increased the bacterial richness index (Chao index, *P* = 0.01), and this effect was more pronounced in grazed plots. One possible explanation is that nutrient inputs from livestock excreta synergistically interacted with warming-induced nutrient release, providing more available nitrogen and thereby improving the survival conditions for bacteria to some extent. As copiotrophic microbes with short generation times, bacteria can rapidly respond to nutrient pulses, leading to a significant increase in richness with enhanced nitrogen supply (Fierer et al., 2007; Ho et al., 2017; Stone et al., 2023).

In contrast, fungal α-diversity indices showed an overall declining trend under the warming treatment. This differential response of increased bacterial richness and decreased fungal diversity may reflect fundamental differences in substrate utilization strategies and environmental sensitivities between the two groups. Bacteria, as copiotrophs with short generation times, can rapidly respond to the nutrient pulses released by warming, swiftly increasing in species number. Fungi, on the other hand, explore soil spaces through hyphal networks and secrete extracellular enzymes to decompose complex organic matter. Key physiological processes such as hyphal extension, spore germination, and extracellular enzyme secretion are highly dependent on soil moisture conditions (Chen et al., 2023; Wang et al., 2021; Donhauser et al., 2023). Warming intensifies soil water evaporation, potentially adversely affecting these moisture-dependent processes in fungi, leading to a decline in their diversity. Furthermore, the preference of fungi for complex organic substrates means their growth is not directly reliant on short-term fluctuations in readily available nitrogen, but rather is more governed by stable moisture conditions and organic matter composition, placing them at a relative disadvantage under the resource changes induced by warming (Zhou et al., 2023).

Furthermore, under the combined effect of warming and heavy grazing, the relative abundance of Pseudomonadota significantly increased. Pseudomonadota are copiotrophic bacteria, a group sensitive to changes in available soil nutrients. Warming accelerated soil organic matter mineralization, increasing the availability of carbon and nitrogen substrates (Wang et al., 2020), thereby promoting the proliferation of Pseudomonadota (Yang et al., 2024; Risch et al., 2019; Meng et al., 2019).

### 4.3 Available nutrients as the primary driver of community variation

This study revealed through both RDA and Pearson correlation analysis that DN and DOC/DN, rather than pH which is common in traditional studies, were the primary environmental factors driving microbial community variation. This may be related to the unique habitat of this experimental site. The soil pH at the study site remains perennially within a narrow range of 9–10. Microbial communities may have adapted to this high-pH environment, so pH is no longer a sensitive factor limiting community change (Zhang et al., 2021; Delgado-Baquerizo et al., 2016). In contrast, such saline-alkaline soils are characterized by a stoichiometric imbalance where nitrogen availability is low relative to carbon, making nitrogen a potentially limiting factor for microbial growth. Nitrogen is an essential element for microbial synthesis of proteins and nucleic acids, and its availability directly constrains microbial growth rates and community assembly (Mooshammer et al., 2014; Cui et al., 2018). In this context, the observation that warming treatment somewhat increased soil DN content (*P* = 0.05, Figure 1e), even if only a marginally significant change, could be sufficient to exert an important influence on microbial communities in a nitrogen-deficient soil environment. While the DOC/DN ratio did not reach statistical significance in univariate analysis, it was identified as an important factor explaining community variation in RDA, indicating that even subtle fluctuations in nutrient stoichiometry have non-negligible ecological significance in nutrient-limited saline-alkaline habitats (Qiang et al., 2024; Diao et al., 2022).

Correlation analysis revealed that bacterial richness and diversity indices were significantly positively correlated with DN and significantly negatively correlated with DOC/DN. This suggests that bacterial richness and diversity increase with rising levels of available soil nitrogen; however, when the DOC/DN ratio is high, this nutrient imbalance may limit bacterial growth. Bacteria, as copiotrophic organisms, prefer to utilize readily decomposable active organic carbon and available nitrogen, and their growth metabolism is highly dependent on the direct supply of nitrogen (Leff et al., 2015). Therefore, in this nitrogen-limited saline-alkaline grassland ecosystem, even a small improvement in nitrogen availability can significantly promote the bacterial community. In contrast, fungi can explore a wider range of soil space through hyphal networks and secrete extracellular enzymes to decompose complex organic matter, making them relatively less dependent on a single nitrogen source (Yang et al., 2021). This may be an important reason why fungal communities were less sensitive than bacteria to changes in DN. This result aligns with the finding from RDA that DN and DOC/DN contributed most to the bacterial community, further supporting the key role of nutrient stoichiometry, particularly the importance of nitrogen availability, in mediating the structure of soil microbial communities in saline-alkaline grasslands (Li et al., 2024).

### 4.4 Changes in co-occurrence network complexity

Grazing disturbance was associated with changes in the topological complexity of microbial co-occurrence networks. Fungal networks under grazing treatments showed a reduction in the number of edges compared to the no-grazing treatment. Disturbances caused by grazing, such as vegetation removal and surface trampling, may disrupt the original potential associations among microorganisms (Liu et al., 2024; Zhao et al., 2025). The bacterial network exhibited higher topological complexity under heavy grazing, which might be related to the release of nutrient resources from increased livestock excreta inputs (Khatri-Chhetri et al., 2024).

The effect of warming on network topology was relatively limited and influenced bacterial and fungal networks in different directions. The fungal network showed a decrease in the average clustering coefficient under warming, indicating that although warming promoted the emergence of more edges, these connections were more topologically dispersed. This adjustment may be related to the sensitivity of fungi to water conditions (Wei et al., 2024b; Yuan et al., 2021).

### 4.5 Study limitations

First, this study had only 4 independent replicate plots per treatment combination, and the sample size may have limited the statistical power to detect smaller treatment effects. This is a plausible explanation for some non-significant results, including the non-significant effects of grazing on most soil physicochemical properties and the non-significant PERMANOVA results for community structure. Future studies employing larger sample sizes or longer experimental durations will help validate and extend the findings of this study. Second, although RDA and Pearson correlation analysis identified DN and DOC/DN as factors strongly correlated with microbial community variation, the observational nature of this field study precludes definitive causal inferences regarding environmental drivers. The relationships observed between environmental variables and microbial communities should be interpreted as statistical associations rather than mechanistic causality.

There was a temporal scale mismatch between the grazing and warming treatments in this study. Grazing had been applied for 8 years cumulatively, while the warming treatment was only implemented for 1 year. Therefore, the observed microbial responses reflect the effect of a short-term warming pulse superimposed on a long-term grazing background. The microbial community may not have yet reached a new equilibrium state under the combined influence of both factors, and the patterns observed in this study may represent transient rather than steady-state responses. The long-term effects of warming on soil microbial communities in saline-alkaline grasslands may differ from the short-term patterns reported here. Co-occurrence networks are constructed based on statistical correlations between microbial abundances and do not represent direct evidence of biological interactions. The observed changes in network topology and complexity should be interpreted as alterations in potential microbial associations rather than confirmed changes in ecological interactions such as mutualism or competition.

The warming subplot area in this study was relatively small (approximately 9 m^2^), constrained by the effective heating area of the infrared warming system. Although soil samples were collected only from a central core area to minimize edge effects, this plot size may limit the representativeness of the warming treatment at the ecosystem scale. Future studies employing larger-scale warming infrastructure will help validate the results reported here.

## 5 Conclusion

This study found that in saline-alkaline grasslands, the interaction between warming and heavy grazing promoted the proliferation of the copiotrophic bacterium Pseudomonadota, suggesting that the combination of increased available nutrients and high-intensity grazing may create favorable conditions for the expansion of specific functional groups. Warming significantly increased the bacterial richness index, whereas fungal α-diversity showed a declining trend under warming, indicating opposing directional responses to warming and reflecting differences between bacteria and fungi in substrate utilization strategies and responses to environmental factors such as moisture. Under grazing treatments, bacterial network topological complexity tended to increase, while fungal network complexity tended to decrease; the effect of warming on network topology was relatively limited. Available nutrients (DN and DOC/DN) were the primary environmental factors driving microbial community variation in this saline-alkaline grassland. Furthermore, long-term grazing may have enhanced microbial community resistance to short-term warming through legacy effects, partially explaining why community structure did not change significantly after warming. Overall, the effect of grazing on microbial communities was relatively limited, while short-term warming, by altering soil nutrient availability and water content, jointly drove the differential responses of bacterial and fungal communities together with grazing disturbance.

## Data Availability

The raw sequencing data and complete metadata files generated in this study have been deposited in the Mendeley Data repository, DOI: 10.17632/g2pfm7nv3x.1. The data are publicly accessible at https://data.mendeley.com/datasets/g2pfm7nv3x/1.
