# Supplementary material for: Effects of short-term warming and grazing on soil microbial communities in saline-alkaline grasslands of northern China
Source: Front Microbiol. 2026 Jun 12;17:1834208. doi: 10.3389/fmicb.2026.1834208 (PMC13303486; doi:10.3389/fmicb.2026.1834208)
Supplement: Supplementary file 1 [file Data_Sheet_1.docx]

Supplementary Material

# Supplementary Figures and Tables

**Table S1.** The effect of warming on the relative abundance of dominant bacterial and fungal phyla under different grazing intensities in soil. Independent samples t-tests were used to compare warming (W) and control (CK) treatments within each grazing intensity (n = 4 independent replicate plots). Data are presented as mean ± SE. Different lowercase letters indicate significant differences between warming treatments at a given grazing intensity (*P* < 0.05). NG: no grazing; LG: light grazing; MG: moderate grazing; HG: heavy grazing; CK: control; W: warming.

**Table S2.** Effects of different grazing intensities on the relative abundance of dominant bacterial and fungal phyla in the control (CK) plots. One-way ANOVA was used to compare grazing intensity treatments (n = 4 independent replicate plots). Data are presented as mean ± SE. Different lowercase letters indicate significant differences among grazing intensities (*P* < 0.05). NG: no grazing; LG: light grazing; MG: moderate grazing; HG: heavy grazing.

**Table S3.** Effects of grazing intensity (G), warming (W) and their interaction (G × W) on soil bacterial and fungal community structures. Permutational multivariate analysis of variance (PERMANOVA) based on Bray-Curtis distances was used (n = 4 independent replicate plots). Data are presented with R², pseudo-F and P values based on 999 permutations.

**Table S4.** Parameters of the co-occurrence network between bacteria and fungi under different grazing intensities and warming conditions (n = 4 independent replicate plots). NG: no grazing; LG: light grazing; MG: moderate grazing; HG: heavy grazing; CK: control; W: warming.

**Figure S1.** Schematic diagram of the experimental design showing the randomized complete block design with four blocks. Each block contained four main plots assigned to four grazing intensity treatments: no grazing (NG), light grazing (LG), moderate grazing (MG), and heavy grazing (HG). Within each main plot, two subplots were randomly assigned to control (CK) and warming (W, +2°C) treatments, resulting in a total of 32 subplots.

**Figure S2** Vegetation canopy warming device (SY-FATI, 800 W, 220 V).

## Supplementary Tables

**Supplementary Table S1.** The effect of warming on the relative abundance of dominant bacterial and fungal phyla under different grazing intensities in soil. Independent samples t-tests were used to compare warming (W) and control (CK) treatments within each grazing intensity (n = 4 independent replicate plots). Data are presented as mean ± SE. Different lowercase letters indicate significant differences between warming treatments at a given grazing intensity (*P* < 0.05). NG: no grazing; LG: light grazing; MG: moderate grazing; HG: heavy grazing; CK: control; W: warming.

| **Treatment** | | **Bacteria** | | | | |  | **Fungi** | | |
| --- | --- | --- | --- | --- | --- | --- | --- | --- | --- | --- |
|  |  | **Pseudomonadota** | **Actinomycetota** | **Acidobacteriota** | **Chloroflexota** | **others** |  | **Ascomycota** | **unclassified_k__Fungi** | **others** |
| **NG** | **CK** | 7.11±0.96a | 6.46±0.45a | 3.58±1.07a | 2.64±0.34a | 1.09±0.01a |  | 19.52±1.34a | 2.18±0.83a | 0.88±0.41a |
|  | **W** | 6.51±0.98a | 6.34±1.02a | 3.81±0.81a | 2.95±0.43a | 1.09±0.31a |  | 18.61±2.08a | 1.73±0.62a | 1.46±1.91a |
| **LG** | **CK** | 6.52±0.98a | 5.70±0.19a | 3.61±1.09a | 2.94±0.58a | 1.38±0.06a |  | 20.40±2.13a | 1.83±1.67a | 0.74±0.54a |
|  | **W** | 7.30±1.02a | 5.76±0.77a | 3.44±1.43a | 2.49±0.53a | 1.42±0.26a |  | 17.90±4.42a | 2.39±0.87a | 1.65±2.60a |
| **MG** | **CK** | 6.52±1.66a | 5.54±0.57a | 3.84±0.63a | 3.17±0.76a | 1.31±0.26a |  | 20.15±2.55a | 2.33±1.10a | 0.61±0.22a |
|  | **W** | 6.13±1.05a | 5.89±0.69a | 3.63±0.61a | 3.04±0.78a | 1.39±0.25a |  | 17.34±4.45 | 1.21±0.72a | 2.93±5.01a |
| **HG** | **CK** | **5.91±0.56a** | 5.86±0.70a | 4.20±0.34a | 3.15±0.31a | 1.26±0.19a |  | 18.17±3.39a | 3.28±2.49a | 0.88±0.31a |
|  | **W** | **7.42±1.09b** | 6.33±0.99a | 3.13±0.91a | 2.59±0.53a | 1.18±0.28a |  | 20.66±1.95a | 1.84±1.09a | 0.84±0.55a |

**Supplementary Table S2.** Effects of different grazing intensities on the relative abundance of dominant bacterial and fungal phyla in the control (CK) plots. One-way ANOVA was used to compare grazing intensity treatments (n = 4 independent replicate plots). Data are presented as mean ± SE. Different lowercase letters indicate significant differences among grazing intensities (*P* < 0.05). NG: no grazing; LG: light grazing; MG: moderate grazing; HG: heavy grazing.

| **Treatment** | **Bacteria** | | | | |  | **Fungi** | | |
| --- | --- | --- | --- | --- | --- | --- | --- | --- | --- |
|  | **Pseudomonadota** | **Actinomycetota** | **Acidobacteriota** | **Chloroflexota** | **others** |  | **Ascomycota** | **unclassified_k__Fungi** | **others** |
| **NG** | 6.81±0.95a | 6.40±0.73a | 3.69±090a | 2.79±0.90a | **1.09±0.40b** |  | 19.06±1.69a | 1.96±0.72a | 1.17±1.31a |
| **LG** | 6.91±1.02a | 5.73±0.52a | 3.52±1.18a | 3.52±1.18a | **2.72±0.57a** |  | 19.15±3.48a | 2.11±1.27a | 1.20±1.80a |
| **MG** | 6.33±1.30a | 5.72±0.62a | 3.74±0.58a | 3.74±0.58a | **3.10±0.72ab** |  | 18.74±3.68a | 1.77±1.05a | 1.77±3.51a |
| **HG** | 6.66±1.14a | 6.09±0.83a | 3.67±0.86a | 3.67±0.86a | **2.87±0.50ab** |  | 19.41±2.89a | 2.56±1.94a | 0.86±0.42a |

**Supplementary Table S3.** Effects of grazing intensity (G), warming (W) and their interaction (G × W) on soil bacterial and fungal community structures. Permutational multivariate analysis of variance (PERMANOVA) based on Bray-Curtis distances was used (n = 4 independent replicate plots). Data are presented with R², pseudo-F and P values based on 999 permutations.

| **Treatment** | **Bacteria** | | | **Fungi** | | |
| --- | --- | --- | --- | --- | --- | --- |
|  | **R^2^** | **F** | **P** | **R^2^** | **F** | **P** |
| **G** | 0.13 | 1.38 | 0.10 | 0.12 | 1.19 | 0.19 |
| **W** | 0.04 | 1.24 | 0.23 | 0.03 | 0.85 | 0.59 |
| **G×W** | 0.07 | 0.76 | 0.85 | 0.07 | 0.73 | 0.90 |

**Supplementary Table S4.** Parameters of the co-occurrence network between bacteria and fungi under different grazing intensities and warming conditions (n = 4 independent replicate plots). NG: no grazing; LG: light grazing; MG: moderate grazing; HG: heavy grazing; CK: control; W: warming.

| **Microbial**  **community** | **Treatment** | **Average**  **degree** | **Average clustering coefficient** | **Average**  **path**  **length** | **Negative** | **Positive** |
| --- | --- | --- | --- | --- | --- | --- |
| **Bacteria** | NG | 10.08 | 0.40 | 4.22 | 1426（62.19%） | 867（37.81%） |
|  | LG | 12.24 | 0.43 | 4.20 | 1516（54.32%） | 1275（45.68%） |
|  | MG | 8.77 | 0.42 | 4.45 | 1183（59.15%） | 817（40.85%） |
|  | HG | 14.79 | 0.43 | 4.22 | 1717（50.93%） | 1654（49.07%） |
|  | CK | 1.29 | 0.31 | 5.35 | 173（58.64%） | 122（41.36%） |
|  | W | 1.47 | 0.33 | 5.08 | 198（59.1%） | 137（40.9%） |
| **Fungi** | NG | 5.38 | 0.50 | 4.78 | 545（83.72%） | 106（16.28%） |
|  | LG | 4.04 | 0.63 | 8.25 | 387（83.77%） | 75（16.23%） |
|  | MG | 1.59 | 0.61 | 7.08 | 461（84.90%） | 82（15.10%） |
|  | HG | 3.89 | 0.49 | 7.25 | 335（75.79%） | 107（24.21%） |
|  | CK | 0.52 | 0.61 | 1.50 | 61（93.85%） | 4（6.15%） |
|  | W | 0.70 | 0.43 | 3.07 | 85（95.51%） | 4（4.49%） |

## Supplementary Figures

**Supplementary Figure S1.** Schematic diagram of the experimental design showing the randomized complete block design with four blocks. Each block contained four main plots assigned to four grazing intensity treatments: no grazing (NG), light grazing (LG), moderate grazing (MG), and heavy grazing (HG). Within each main plot, two subplots were randomly assigned to control (CK) and warming (W, +2°C) treatments, resulting in a total of 32 subplots.


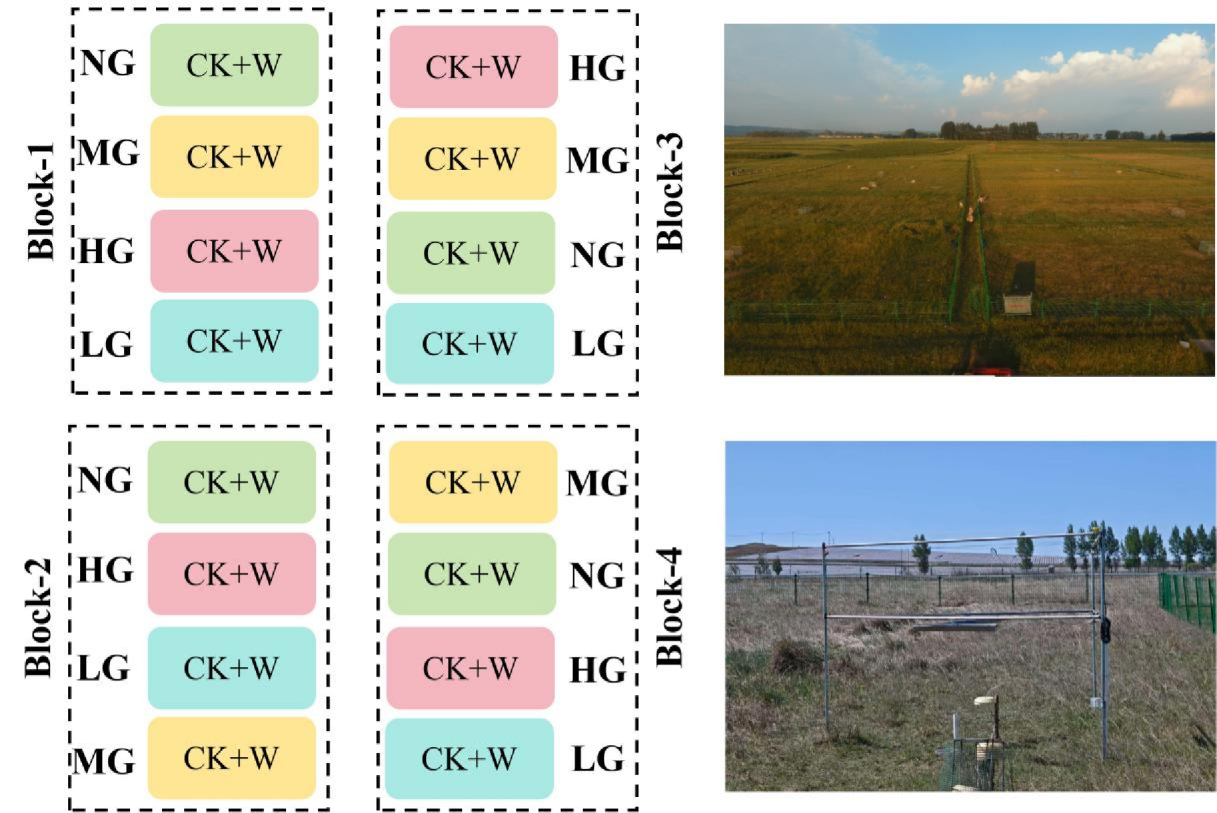


**Supplementary Figure S2.** Vegetation canopy warming device(SY-FATI, 800 W, 220 V).

**
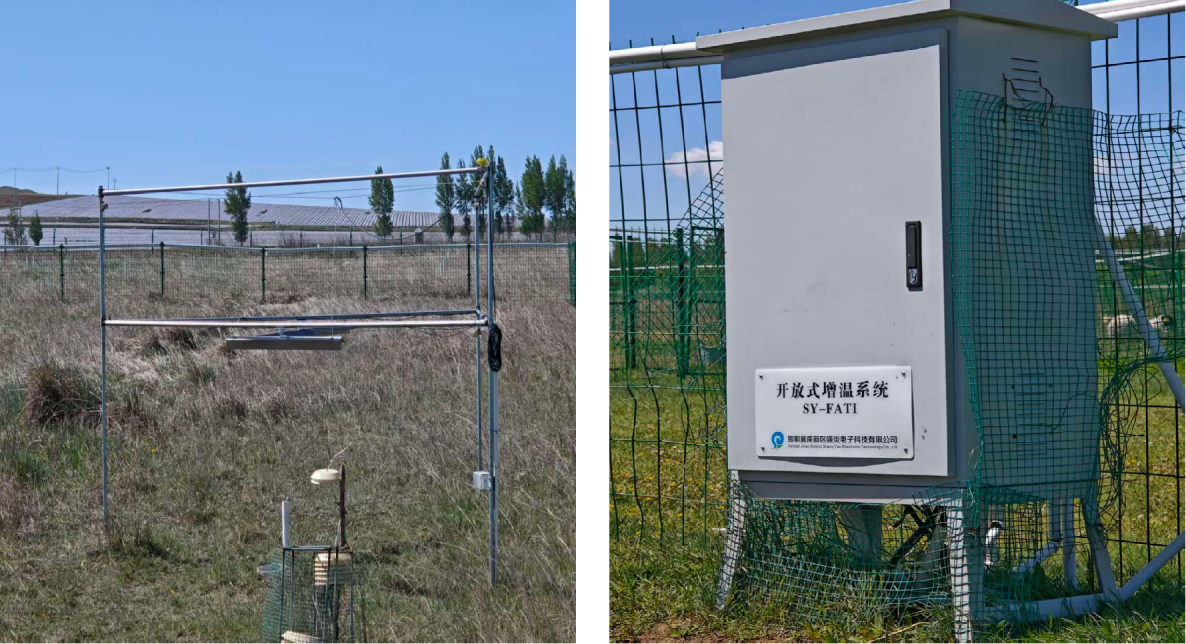
**
